# Electrospun Poly(l-lactide)/Poly(ethylene glycol) Scaffolds Seeded with Human Amniotic Mesenchymal Stem Cells for Urethral Epithelium Repair

**DOI:** 10.3390/ijms17081262

**Published:** 2016-08-09

**Authors:** Xiaokui Lv, Qianping Guo, Fengxuan Han, Chunyang Chen, Christopher Ling, Weiguo Chen, Bin Li

**Affiliations:** Departments of Urology and Orthopaedic Surgery, The First Affiliated Hospital, Orthopaedic Institute, Soochow University, 708 Renmin Rd., Suzhou 215007, China; xiaokui86@163.com (X.L.); guoqianping@suda.edu.cn (Q.G.); fxhan@suda.edu.cn (F.H.); 18862238857@163.com (C.C.); christopherwfling@gmail.com (C.L.)

**Keywords:** human amniotic mesenchymal cells, electrospinning, urethral defect, tissue engineering, poly(l-lactide), poly(ethylene glycol)

## Abstract

Tissue engineering-based urethral replacement holds potential for repairing large segmental urethral defects, which remains a great challenge at present. This study aims to explore the potential of combining biodegradable poly(l-lactide) (PLLA)/poly(ethylene glycol) (PEG) scaffolds and human amniotic mesenchymal cells (hAMSCs) for repairing urethral defects. PLLA/PEG fibrous scaffolds with various PEG fractions were fabricated via electrospinning. The scaffolds were then seeded with hAMSCs prior to implantation in New Zealand male rabbits that had 2.0 cm-long defects in the urethras. The rabbits were randomly divided into three groups. In group A, hAMSCs were grown on PLLA/PEG scaffolds for two days and then implanted to the urethral defects. In group B, only the PLLA/PEG scaffolds were used to rebuild the rabbit urethral defect. In group C, the urethral defect was reconstructed using a regular urethral reparation technique. The repair efficacy was compared among the three groups by examining the urethral morphology, tissue reconstruction, luminal patency, and complication incidence (including calculus formation, urinary fistula, and urethral stricture) using histological evaluation and urethral radiography methods. Findings from this study indicate that hAMSCs-loaded PLLA/PEG scaffolds resulted in the best urethral defect repair in rabbits, which predicts the promising application of a tissue engineering approach for urethral repair.

## 1. Introduction

Urethral defects caused by urethral trauma, congenital malformation, and tumor are common causes of urological surgeries [1]. While regular defects can be repaired using end-to-end anastomosis or buccal mucosa tissues of patient, repairing larger segmental urethral defects (longer than 2 cm) remains challenging [2,3,4]. Encouraged by the success of a few recent clinical studies, tissue engineering-based urethral reconstruction is believed to be a promising treatment for urethral defect repair [5,6,7,8,9]. Three major approaches which involve cells alone, scaffolds alone, and cell-seeded scaffolds, respectively, have been used in urethral tissue engineering. Among them, cell-seeded scaffolds may lead to the most effective urethral regeneration [2,10,11].

To date, a variety of scaffold materials have been used for urethral tissue engineering. These include: (1) natural materials such as collagen, hyaluronic acid, and alginate; (2) biological matrices derived from decellularized tissues from the foreskin, bladder mucosa, small intestinal submucosa, and tunica vaginalis; and (3) synthetic polymers such as polyglycolic acid (PGA), polylactide (PLA), poly(l-lactide-*co*-glycolide) (PLGA), and polycaprolactone (PCL). Among them, natural materials are often of insufficient strength to support urethral repair [12]. Decellularized tissues, while containing cell signaling molecules which may favor tissue regeneration, suffer from source shortages and high complication rates [3,4,13]. Synthetic biodegradable polymers, on the other hand, have been widely used in tissue engineering [14,15]. A commonly used polymer, poly(l-lactide) (PLLA) possesses good mechanical properties and excellent biocompatibility. In order to achieve moderate hydrophilicity [16,17], PLLA can be blended with hydrophilic polymers such as poly(ethylene glycol) (PEG). Such a composite scaffold has been shown to possess adequate hydrophilicity to support cell adhesion and proliferation [18].

Cells are another critical component for urethral tissue engineering. Being the most important component of the urinary tract, epithelial cells have been used for urethral regeneration. However, the sources of urethral epithelial cells are limited. Harvesting these tissues involves a complicated operation to the genitourinary tract [2]. In addition, the proliferation of urothelial cells is limited. In contrast, mesenchymal stem cells (MSCs) which have multi-potential differentiation and self-renewal ability, including bone marrow mesenchymal stem cells (BMSCs) and adipose-derived mesenchymal stem cells (ADMSCs), are believed to be ideal cell sources for urethral tissue engineering [19,20,21]. However, the applications of such autologous adult MSCs are also limited due to the painful and invasive harvest procedure, limited number, and loss of stemness during in vitro expansion [22]. Recently, human amniotic mesenchymal stem cells (hAMSCs), which are derived from the amniotic membrane, have drawn much attention [23]. Similar to BMSCs and ADMSCs, hAMSCs can be induced into many lineages such as adipocytes, osteocytes, chondrocytes, and endothelial cells [24,25]. In addition, hAMSCs have strong proliferation capacity in vitro and are immunologically tolerant [26]. A major advantage of hAMSCs is their ready availability, which eliminates the invasive procedures and ethical concerns of cell harvesting [23]. Therefore, hAMSCs may be a good candidate cell source in urethral tissue engineering.

In this study, we prepared fibrous PLLA/PEG composite scaffolds via electrospinning, a technique which enables the fabrication of highly porous structures with the diameter of fibers ranging from a few hundred nanometers to several microns [27,28]. We then cultured hAMSCs on the scaffolds to form cells-scaffold constructs in vitro, which we hypothesized would facilitate the repair of urethral defects. Following that, the cells-scaffold constructs were implanted to the urethral defects of rabbits to examine their repair capability. After up to three months from implantation, the urethral morphology, tissue reconstruction, luminal patency, and complication incidence of animals were checked using histological evaluation and urethral radiography approaches.

## 2. Results

### 2.1. Fabrication of Poly(l-lactide)/Poly(ethylene glycol) (PLLA/PEG) Scaffolds

**Figure 1:**
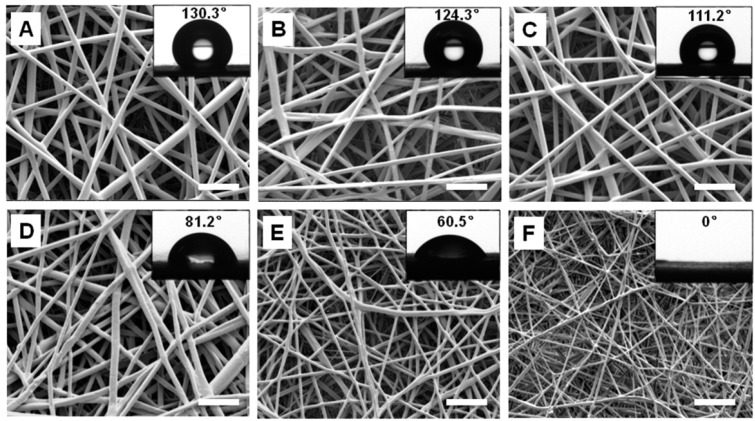
SEM and water contact angle measurements of electrospun poly(l-lactide)/poly(ethylene glycol) (PLLA/PEG) scaffolds with PEG fractions of 0% (**A**); 10% (**B**); 20% (**C**); 30% (**D**); 40% (**E**); and 50% (**F**), respectively. Both the fiber diameter and water contact angle decreased with the increase of PEG content. Scale bars, 10 μm.

Fibrous scaffolds of PLLA/PEG composites were fabricated using electrospinning technique. As can be seen from the SEM images, the fiber diameter decreased with an increase of PEG fraction in the composites (Figure 1). The average fiber diameters of PLLA, PEG10, PEG20, PEG30, PEG40, and PEG50 samples were 1.6 ± 0.21, 1.5 ± 0.41, 1.48 ± 0.52, 1.3 ± 0.38, 0.9 ± 0.3, and 0.5 ± 0.24 μm, respectively. The scaffolds did not show apparent deformation upon immersion in cell culture medium, indicating that they had good dimensional stability.

The wettability of PLLA/PEG scaffolds was determined using water contact angle measurement. Apparently, the hydrophilicity of the membrane was improved by increasing PEG content. The average water contact angle of PLLA, PEG10, PEG20, PEG30, PEG40, and PEG50 were 130.3 ± 2.1°, 124.8 ± 2.5°, 111.2 ± 3.1°, 76.6 ± 4.9°, 60.3 ± 2.8°, and 0°, respectively (Figure 1). When the PEG fraction reached 30%, the membrane became hydrophilic (i.e., water contact angle < 90°). Further increasing the PEG fraction to 50% resulted in the formation of super-hydrophilic membranes (i.e., water contact angle = 0°).

**Figure 2:**
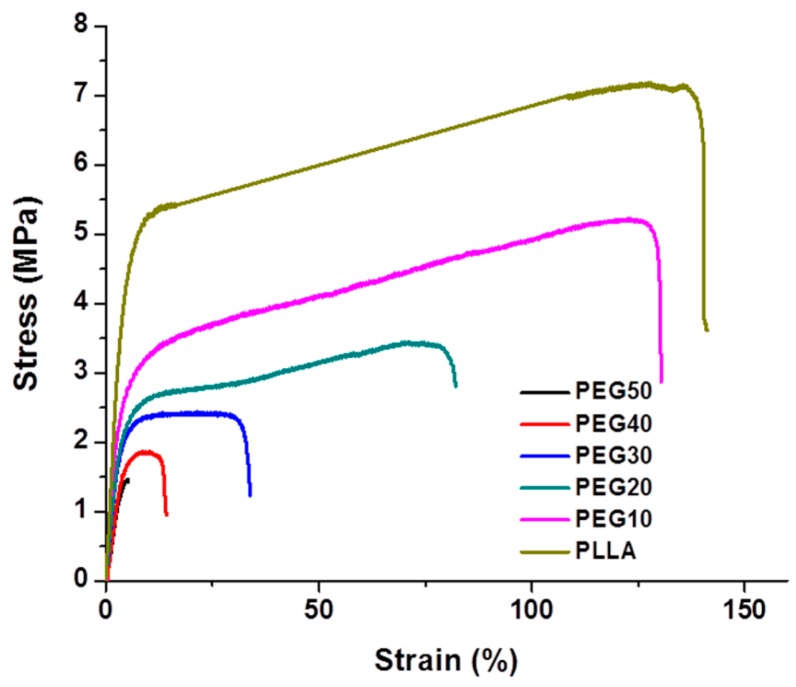
Stress-strain curves of electrospun PLLA/PEG scaffolds with various PEG fractions.

The mechanical properties of electrospun PLLA/PEG scaffolds were determined using tensile tests (Figure 2). Among all the composite scaffolds, the PEG10 sample showed the best mechanical properties. Increasing the PEG fraction in the composites resulted in deterioration of their mechanical characteristics. For example, the tensile strength, Young’s modulus, and elongation of PEG10 samples were 5.3 ± 0.19 MPa, 132.8 ± 1.56 MPa, and 126.0% ± 14.26% respectively. However, the tensile strength, Young’s modulus, and elongation of PEG50 samples dropped to 1.4 ± 0.10 MPa, 31.4 ± 3.12 MPa, and 4.0% ± 1.16%, respectively.

### 2.2. Isolation and Characterizations of Human Amniotic Mesenchymal Cells (hAMSCs)

**Figure 3:**
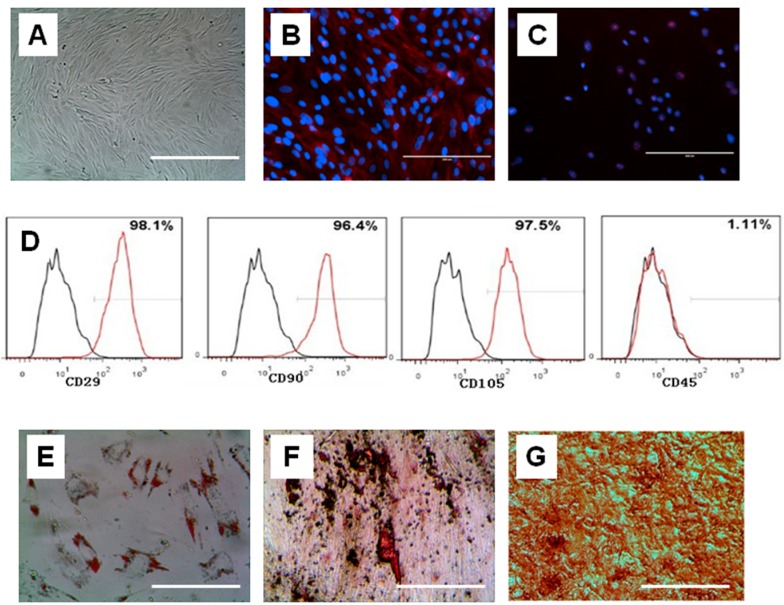
Identification of human amniotic mesenchymal cells (hAMSCs). (**A**) The fibroblast-like morphology of hAMSCs at P3; (**B**,**C**) hAMSCs were positive for stem cell markers Oct-4; (**B**) and SSEA-4 (**C**); (**D**) Immunophenotypical characterization of hAMSCs. Cells at the 5th culture passage were trypsinized, labeled with antibodies against the antigens indicated and analyzed by flow cytometry. hAMSCs expressed CD29, CD90 and CD105, but did not express CD45. Black, isotype control; red, antibody; (**E**–**G**) Multi-lineage differentiations of hAMSCs in vitro. hAMSCs were stained with Oil Red O after being induced in adipogenic differentiation medium for 2 weeks (**E**). Small colonies with lipid secretion were clearly seen. hAMSCs were stained with Alizarin red S after being induced in osteogenic differentiation medium for 3 weeks (**F**). hAMSCs were stained Safranin O after being induced in chondrogenic differentiation medium for 3 weeks (**G**). Scale bars, (**A**–**C**) 200 µm; (**E**–**G**) 50 µm.

hAMSCs were isolated using a combined trypsin-collagenase method and cultured in Dulbecco’s Modified Eagle’s medium (DMEM) medium supplemented with 10% fetal bovine serum (FBS). After 24 h, hAMSCs adhered to the plate and started to proliferate. The morphology of the hAMSCs was fibroblast-like, polygonal, or round after 48 h. Non-adhered cells were removed by changing the medium. Cells formed colonies 7 days later and reached 80%–85% confluence after 14 days. The cells were passaged every 3–4 days after P0 and could be passaged up to P15 without apparent morphological change. After passage, the cell morphology was fibroblast-like (Figure 3A).

In order to identify the hAMSCs, cells at passage 3 were assayed by immunofluorescence and flow cytometry. The cells were stained positive for stem cell markers such as Oct-4 and nucleostemin (Figure 3B,C). In flow cytometry analysis, the cells showed expression of classic MSC surface markers CD29, CD90 and CD105. In addition, they also slightly expressed CD45 (Figure 3D).

The multi-differentiation potential of hAMSCs was checked in vitro for adipogenesis, osteogenesis, and chondrogenesis. The results showed that hAMSCs were positive by Oil Red O staining, indicating that they secreted oil after adipogenic induction (Figure 3E). After osteogenic differentiation, the cells were then treated with Alizarin Red S solution to test for calcium deposits. The positive and strong staining of Alizarin Red S indicated osteogenesis (Figure 3F). The production of sulfated proteoglycan, as shown by Saffranin O staining solution, confirmed that the hAMSCs had undergone chondrogenesis (Figure 3G).

### 2.3. Biocompatibility of PLLA/PEG Scaffolds

**Figure 4:**
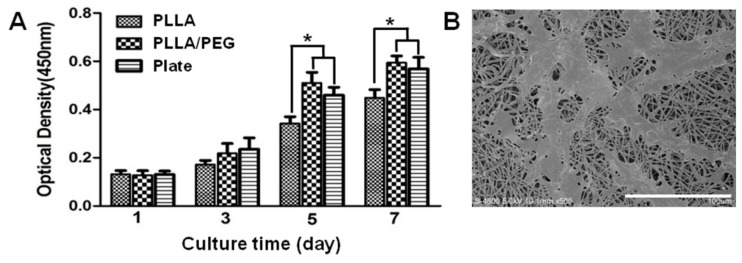
Proliferation (**A**) and SEM images (**B**) of hAMSCs on electrospun PLLA/PEG scaffolds. * *p* < 0.05. Scale bar, 100 μm.

In order to test the cytotoxicity of PLLA/PEG scaffolds, the proliferation of hAMSCs on the scaffolds was examined using Cell Counting Kit-8 (CCK-8) assays. The cells were cultured on a plate and a PLLA scaffold as the control. As seen, the PLLA/PEG scaffolds had little cytotoxicity and well sustained hAMSC proliferation (Figure 4A). Compared to pure PLLA, hAMSCs appeared to proliferate faster on PLLA/PEG scaffolds. In addition, the SEM images showed that many fibroblast-like hAMSCs spread on the scaffolds with some of the pseudopodia approaching the inner part of scaffolds, again indicating the biocompatibility of PLLA/PEG scaffolds (Figure 4B).

### 2.4. Urethrography Analysis and Morphological Observation

**Figure 5:**
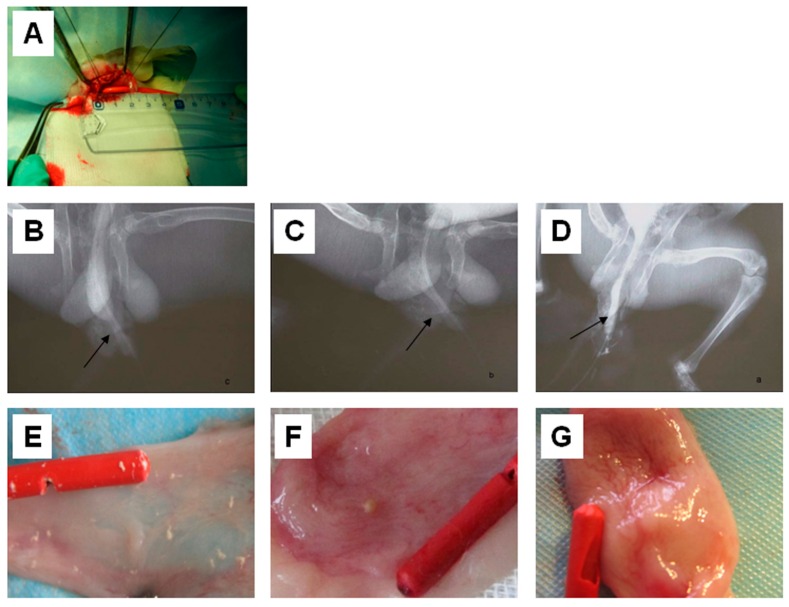
Implantation of electrospun PLLA/PEG scaffolds and human amniotic mesenchymal cells (hAMSCs) in urethral defects of rabbits. (**A**) A urethral mucosa defect of about 2 × 1.5 cm was formed in a rabbit; (**B**–**D**) Retrograde urethrograms of the rabbits that were subjected to a mock operation (**B**), implanted with PLLA/PEG scaffold (**C**); and hAMSCs–PLLA/PEG construct (**D**); respectively; (**E**–**G**) Gross observation of mucosa healing and calculus formation in the rabbits that were subjected to a mock operation (**E**); implanted with PLLA/PEG scaffold (**F**); and hAMSCs–PLLA/PEG construct (**G**); respectively.

All rabbits had a retrograde urethrogram (RUG) before the surgery (Figure 5A) and showed normal morphology. After the operations, the RUG images indicated that strictures occurred in both group B and group C (Figure 5B,C). However, no signs of stricture and fistula were found in the group A at 12 weeks post-operation (Figure 5D). As shown in Table 1, the incidence of complications in the control group (group C) was 72.22% (13/18). This was significantly higher than the incidence of complications in group A (0%) and group B (5.55%). However, there was no statistically significant difference between the complication incidences of group A and group B.

The urethral specimens were harvested and observed 4 weeks after the surgeries (Figure 5E–G). In group C, non-absorbable 4-0 vicryl marking sutures could be found. The urethral defect surface was uneven and covered by a scar. Mucous membrane contraction, luminal stricture, bladder crystallization and a large amount of the urethra were visible (Figure 5E). In group B, a part of the PLLA/PEG scaffold was exposed to the inside of the urethra lumen without a mucous membrane. Compared to group C, the luminal stricture in group B was relieved; however, scar formation, urethra and bladder crystallization was still obvious. In contrast, the implant in group A was covered with a mucous membrane, and no apparent scar or crystallization was observed (Figure 5G).

### 2.5. Histological Evaluation

**Figure 6:**
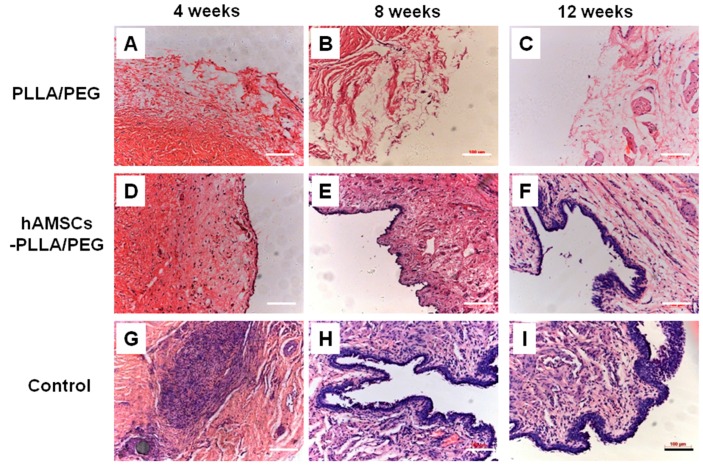
Hematoxylin–eosin (H&E) staining of the urethral tissues at 4, 8, and 12 weeks post-operation. (**A**–**C**) implanted with PLLA/PEG scaffolds; (**D**–**F**) implanted with hAMSCs–PLLA/PEG constructs; (**G**–**I**) given mock operation. Scale bars, 100 μm.

The histological analysis of the urethral specimens at 4, 8, and 12 weeks post-operation is shown in Figure 6. In group B, approximately half of the PLLA/PEG scaffolds degraded after 4 weeks. Some vascellum, collagen tissue, and lymphocytes formed, while no epithelial cells were present until 12 weeks (Figure 6A–C). Compared to group B, PLLA/PEG scaffolds in group A partially degraded and a layer of epithelial cells was observed on the surface of scaffolds. Moreover, the cellular layer increased over time. A multilayered urothelium was present in group A; vascellum, smooth muscle, and fibrous tissues were found to be arranged along the PLLA/PEG fibers (Figure 6D–F). In contrast, there were many lymphocytes invading the defected tissue, and a few vascellums were found 4 weeks after the surgery in group C. The number of cells increased in the defect region, but the cells were in an unorganized formation. In addition, many fibrous tissues containing a few smooth muscle fibers formed after 6 weeks. The urethral mucosa was still discontinuous, and some lymphocytes and fibrous tissue existed in the defect tissue after 8 weeks (Figure 6G–I).

**Figure 7:**
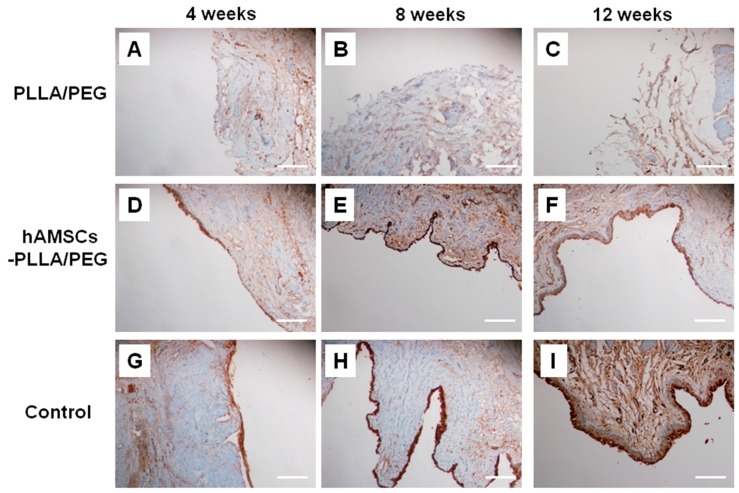
Immunohistochemical evaluation of urethral tissues by AE1/AE3 staining at 4, 8, and 12 weeks post-operation. (**A**–**C**) implanted with PLLA/PEG scaffolds; (**D**–**F**) implanted with hAMSCs–PLLA/PEG constructs; (**G**–**I**) given mock operation. Scale bars, 100 μm.

Further, the urethral epithelial cells were identified using a mouse anti-human cytokeratins AE1/AE3 monoclonal antibody. The results were negative in group B, indicating that no epithelial layer formed (Figure 7A–C). However, group A was positive for AE1/AE3 staining, indicating the presence of multilayered urothelium in the PLLA/PEG scaffold (Figure 7D–F). In the control group C, the thickness of the epithelial cell layer increased gradually from 4 to 12 weeks, and the cell layers became more and more uniform (Figure 7G–I).

## 3. Discussion

Repair of long segmental urethral defects remains technically challenging. In this study, a tissue-engineering approach which combined the use of electrospun fibrous PLLA/PEG scaffolds and hAMSCs was explored for urethral defect repair in rabbits. A widely used technology for fabricating nano-/microfibrous scaffolds, electrospinning possesses a number of advantages [27,29]. First, the electrospun scaffolds have high porosity and complete interconnectivity, which is necessary for cell migration, nutrient diffusion, and vascellum in-growth. Second, the nano-/microfibrous structure of scaffolds well mimics the extracellular matrix (ECM) structure of native tissues and favors cell adhesion, proliferation, and differentiation. In a previous study, electrospun silk fibroin scaffolds seeded with urothelial cells were applied to the dorsal urethral mucosa defect of beagles. After up to 6 months, gradual epithelial cell development and stratified epithelial layers were clearly seen in the scaffolds, implying the potential of using electrospun scaffolds for urethral reconstruction [30].

In order to identify the optimal scaffold for urethral tissue engineering, PLLA/PEG scaffolds with different PEG fractions were prepared. The diameter of fibers in the scaffolds was in the range of 500–1500 nm, and it decreased with the increase of PEG content. Since PEG has lower molecular weight compared to PLLA, it may act as a lubricant in the PLLA/PEG mixed solution [18]. Therefore, higher PEG content contributed to thinner fibers as shown in Figure 2. Moreover, the hydrophilicity/hydrophobicity balance of scaffolds is very important for protein adsorption [17,31]. It has been reported that biomaterials with moderate hydrophilicity had better biocompatibility [32,33]. The introduction of PEG improved the hydrophilicity of PLLA scaffolds and consequently led to the faster proliferation of hAMSCs on PLLA/PEG scaffolds (Figure 4). In addition, the mechanical property of scaffolds plays a critical role in tissue engineering. The strength at rupture, Young’s modulus, and elongation of normal rabbit urethra are 0.2 ± 0.07, 1.98 ± 0.73, and 173.67 ± 50.67 MPa, respectively [34]. The addition of PEG dramatically deteriorated the mechanical properties of PLLA/PEG scaffold. Taking into consideration of both the hydrophilicity and mechanical properties of scaffolds, the PEG30 scaffold in which a mixture of PLLA and 30% PEG was used for electrospinning was chosen as the scaffolds for the following in vitro and in vivo studies. As shown in Figure 4, hAMSCs adhered and proliferated well on the PLLA/PEG scaffold (PEG30), indicating the possibility of using them for urethral reconstruction in vivo.

In the in vivo tests, the hAMSCs–PLLA/PEG constructs were implanted into the urethral defects of rabbits. Clearly, the incidences of urethral stricture, urinary fistula, and complications associated with implantation using hAMSCs-seeded scaffolds (group A) were markedly lower than the two control groups (groups B and C). In addition, no urethral stricture was observed when hAMSCs-seeded scaffolds were implanted. The histological results also show that in group A, epithelial cells covered the defect gradually and formed multi-layer mucosa membranes which were similar to that of normal urethral tissue after 12 weeks. Further, immuofluoresence analysis revealed that the specimens implanted with hAMSCs-seeded scaffolds were positive in pan-cytokeratin AE1/AE3 staining, indicating the presence of urethral epithelial cells. Since urethral epithelial cells played an important role in protecting the underlying muscle tissues from the caustic properties of urine, urethral epithelium regeneration is critical for urethra reconstruction [35]. In this study, the urethral epithelial cells attached and formed multiple layers on hAMSCs–PLLA/PEG scaffold. In contrast, no urethral epithelium was seen in the specimens from the group using PLLA/PEG scaffold alone (group B). Together, these results indicate that PLLA/PEG scaffold supported the growth of epithelial cells, and the presence of hAMSCs promoted the regeneration of urethral epithelium. 

## 4. Materials and Methods

### 4.1. Materials 

Twenty-seven male New Zealand white rabbits (3 month-old, 2.5–3.0 kg) were used in the experiment. Fetal bovine serum (FBS), 0.25% penicillin-streptomycin, and 0.05% trypsin contained 0.53 mM ethylenediaminetetraacetic acid (EDTA) were all purchased from Gibco (Grand Island, NY, USA). Dulbecco’s Modified Eagle’s medium (DMEM) was supplied by Hyclone (Logan City, UT, USA). PLLA (${\bar{M}}_{w}$ = 50 kDa) and PEG were provided by Daigang Biotechnology Company (Jinan, China) and Yarebio (Shanghai, China), respectively. The antibodies which included anti-CD29, CD45, CD90 and CD105 were purchased from Neomarkers (Thermo Fisher Lab Vision, Fremont, CA, USA).

### 4.2. Preparation of PLLA/PEG Scaffolds Using Electrospinning

To prepare the PLLA/PEG fibers, a mixture of PLLA and PEG with various PEG fractions (0%, 10%, 20%, 30%, 40%, and 50%) was dissolved in chloroform/dimethylformamide (*v*/*v* = 7:3) to obtain an electrospinning solution. Five milliliters of the electrospun solution was fed into a plastic syringe fitted with a stainless-steel blunt needle of 0.21 mm in diameter and placed in the electrospinning machine. The solution was electrospun at a 0.5 mL/h flow rate, a voltage of 16.9 kV and the distance between the collection and capillary tip was 15–25 cm. The obtained fibers were recorded as PLLA, PEG10, PEG20, PEG30, PEG40, and PEG50, respectively. The morphology of the electrospun fibers was characterized by scanning electron microscope (SEM, S-4800, Hitachi, Tokyo, Japan) and the fiber diameter was measured from the SEM images. The hydrophilicity of the membrane was characterized via water contact angle measurement. For further applications in urethral tissue engineering, the mechanical properties of the scaffolds were tested in this experiment. The membrane with a thickness in the range of 0.2–0.4 mm was cut into 15 × 3 mm rectangles and the tensile tests of the electrospun membrane were performed using a tensile tester. The stress-strain curves of the samples were obtained from the load-deformation curves recorded at a cross-head speed of 5 mm/min.

### 4.3. Isolation of hAMSCs

Fresh amniotic membrane was obtained from a maternal donor at the First Affiliated Hospital of Soochow University, Suzhou, China. Informed consent was given by the participant and the procedure for hAMSCs isolation was approved by the ethics committee of the hospital. The tissue was tested to be negative for Hepatitis A, Hepatitis B, HCV, HIV, syphilis, and influenza. After the blood was washed away by phosphate buffer solution (PBS), the amniotic membrane was separated from the chorion. hAMSCs were then isolated from the digesting amniotic membrane with combined trypsin-collagenase method and cultured in DMEM supplemented with 10% FBS.

### 4.4. Characterizations of hAMSCs

The cell phenotype was characterized by differentiation and immunofluorescence measurements. hAMSCs at passage 0–3 were used for immunofluorescence. Cells were washed three times with PBS and fixed in cold 4% paraformaldehyde for 15 min. Following that, the cells were washed twice with PBS and treated with methanol at −20 °C for 5 min. For Oct-4 immunofluorescence, the fixed cells were washed with PBS three times and blocked with 4% BSA for 30 min before being incubated with mouse anti-human Oct-4 antibody (Cat. # MAB4401, Millipore, Billerica, MA, USA; 1:500 dilution in PBS) overnight at 4 °C. After being washed with PBS, the cells were incubated with Cy3-conjugated goat anti-mouse secondary antibody (A10521, Invitrogen, Carlsbad, CA, USA; 1:1000) for 1 h at room temperature. DNA was visualized by DAPI staining and cells were viewed under a fluorescence inverted microscope (EVOS f1, AMG, Carlsbad, CA, USA USA). For nucleostemin staining, goat anti-human nucleostemin antibody (GT15050, Neuromics, Edina, MN, USA; 1:250) along with Cy3-conjugated donkey anti-goat IgG secondary antibody (AP180C, Millipore; 1:1000) were used.

hAMSCs at passage 0–3 were used for flow cytometry. The cells were digested by trypsin and collected. After washed with PBS three times, 1 × 10^6^ cells were incubated with the antibody for 30 min and then washed twice with PBS. Analysis was performed after blending.

The differentiation potentials of the hAMSCs were tested in vitro by adipogenic, osteogenic, and chondrogenic differentiation assays. Passage 2–4 hAMSCs were seeded at a density of 4 × 10^4^ cells/well in a 24-well plate in a basic culture medium (DMEM supplemented with 10% FBS and 100 U/mL penicillin). For adipogenesis, cells were induced in an adipogenic medium consisting of basic culture medium supplemented with 0.1 μM dexamethasome (D4902, Sigma, Saint Louis, MO, USA), 1 mM isobutylmethylxanthine (IBMX) (I7018, Sigma), 10 ng/L insulin and 60 mM indomethacin (I7378, Sigma) after reaching full confluence at 2 days. For osteogenesis, cells were induced in an osteogenic medium consisting of a basic culture medium supplemented with 0.1 μM dexamethasone, 0.05 mM ascorbic acid 2-phospate (A8960, Sigma), and 10 mM β-glycerolphosphate (G8981, Sigma) when cells reached 80% confluence. For chondrogenesis, cells were induced in a chondrogenic medium consisting of a basic culture medium supplemented with 40 μg/mL proline (P5607, Sigma), 39 ng/mL dexamethasone, 10 ng/mL TGF-β3 (T5425, Sigma), 50 μg/mL ascorbate 2-phosphate, 100 μg/mL sodium pyruvate (P8574, Sigma), and 50 mg/ml insulin-transferrin-selenious acid mix (ITS) (I1884, Sigma) when the cells were 80% confluent. In the control groups, all the cells were cultured in a basic culture medium. The cell media was changed every three days. Oil Red O, Alizarin Red S and Safranin O staining were used for adipogenesis, osteogenesis and chondrogenesis assays, respectively.

### 4.5. Culture of hAMSCs on PLLA/PEG Scaffolds

MC3T3-E1 mouse preosteoblasts (CRL-2594, subclone 14, ATCC, Rockville, MD, USA) were cultured in α-mininum essential medium (α-MEM, Gibco) with 10% fetal bovine serum (Gibco) and 1% penicillin/streptomycin under 37 °C, 5% CO_2_ environment. Cells were seeded onto different experimental substrates. The plates with dimensions 5.8 mm were placed on 96-well polystyrene plates. The concentration of the cells initially seeded onto the specimen substrate was 1 × 10^4^ cells/well. The 13 mm plates were placed on 24-well polystyrene plates and the concentration was 3 × 10^4^ cells/well. The 31 mm plates were placed on 6-well polystyrene plates and the concentration was 3 × 10^5^ cells/well. In the osteogenic differentiation assay, after the cells were cultured for 24 h in the medium described earlier, 10 mM β-glycerol phosphate, 50 μg/mL ascorbic acid, and 10 nM dexamethasone were added for osteogenic induction. The media were refreshed every three days.

### 4.6. Cell Morphology

PLLA/PEG scaffolds (PEG30) were fixed to 96-well plates or 12-well plates. The scaffolds were sterilized by ^60^Co and immersed in PBS for 10 min before cell seeding. In the cell proliferation assay, hAMSCs were seeded at a density of 1 × 10^4^ cells/well to a 96-well plate and incubated at 37 °C with 5% CO_2_. After culturing for 1, 3, 5, and 7 days, the absorbance was measured according to the CCK-8 kit protocol. For SEM observation, hAMSCs were seeded at a density of 5 × 10^4^ cells/well in a 12-well plate and cultured for 24 h. The cell-scaffold composite was fixed in 4% glutaraldehyde for 4 h and then freeze-dried. The cell morphology was examined via SEM (S-4800, Hitachi).

### 4.7. Preparation of hAMSC-PLLA/PEG Constructs for Implantation

PLLA/PEG scaffolds (PEG30) were cut into 2 × 1.5 cm pieces and sterilized with ^60^Co at a dosage of 12–15 kGy. The pieces were immersed in DMEM medium for 24 h and then transferred to FBS for 12 h. The hAMSCs (passage 3) were seeded at a density of 3–5 × 10^6^ cells/cm^2^ to the scaffold and cultured in DMEM containing 10% FBS at 37 °C with 5% CO_2_ for 48 h to obtain the hAMSC-PLLA/PEG constructs for implantation.

### 4.8. Animal Surgeries

Twenty-seven rabbits were divided into 3 groups (*n* = 9). In Group A, the artificial urethral defects were covered with hAMSCs–PLLA/PEG scaffolds. In Group B, the defects were covered with PLLA/PEG scaffolds. Group C served as a control group in which the animals were subjected to sham surgeries. The rabbits (~3 kg) from all groups were anesthetized using urethane. Through a ventral longitudinal penile-skin incision, a dorsal urethral mucosa segment of about 2 × 1.5 cm which was about 0.5 cm from the external urethral orifice was excised. In group A or B, the hAMSCs–PLLA/PE scaffold or PLLA/PEG scaffold was trimmed and placed over the urethral defect. The urethral repair was continuously sutured with 7-0 vicryl suture. Multiple nonabsorbable 4-0 vicryl sutures were stitched at the four corners of the scaffold and were used as the markers for removing the tissue. In group C, the incision was closed immediately after the corpora cavernosa was blunt dissected. A catheter was inserted into the bladder and sewed with absorbable 5-0 vicryl sutures for 3–5 days. After operation, the rabbits were treated with gentamicin for 5–7 days. The animal surgery protocol was approved by the Institutional Animal Care and Use Committee (IACUC) of Soochow University.

### 4.9. Postoperative Evaluations

Three rabbits were sacrificed at 4, 8, and 12 weeks post-operation. Retrograde urethrograms were performed before and after the sacrifices to observe the situation of urethra. Mucosa healing and calculus formation were observed after the urethral incision. Histological examinations were tested using hematoxylin–eosin (H&E) staining and immunohistochemistry.

### 4.10. H&E Staining

Specimens were fixed in a 10% formalin solution and dehydrated in an ascending series of ethanol and embedded in paraffin. Sections which were 6 μm thick were prepared using a microtome and mounted on subbed glass slides. Slides containing four randomly selected sections from each implant were dipped in hematoxylin for 6 min and then in eosin for 1 min. The stained slides were observed using an inverted phase contrast microscope (Axiovert 200, Carl Zeiss, Oberkochen, Germany).

### 4.11. Immunohistochemistry

The deparaffinized sections of urethra were thoroughly washed and non-specific endogenous peroxidase activity was quenched by immersing in 3% H_2_O_2_/methanol for 15 min. After being washed three times with PBS, the sections on the slides were incubated overnight with a mouse monoclonal pan-Cytokeratin antibody (AE1/AE3) (sc-81714, Santa Cruz, Santa Cruz, CA, USA; 1:500 dilution in PBS) at 4 °C. Slides incubated with isotype matched control antibodies or PBS without primary antibodies were used as negative controls. The slides were then washed with PBS and further incubated with horseradish peroxidase (HRP)-conjugated goat anti-mouse IgG (H+L) antibody (AB503, Novoprotein, Shanghai, China; 1:500 dilution in PBS) at 37 °C for 30 min. Finally, the HRP substrate was applied for visualization.

### 4.12. Statistics Analysis

All quantitative data are presented as mean ± standard deviation with no less than three replicates for each experimental condition. Statistical analyses were performed by SPSS software. Kruskal-Wallis one-way analysis of variance (ANOVA) tests followed by Tukey post hoc tests were used. Unpaired Student’s *t*-tests were also used where appropriate. Difference between the groups is considered statistically significant if *p* is less than 0.05.

## 5. Conclusions

In summary, PLLA/PEG fibrous scaffolds with various PEG contents have been fabricated via electrospinning in this study. The hAMSCs adhered and proliferated well on the scaffolds. After being seeded with hAMSCs, the scaffolds were implanted in the long segmental urethral defects of rabbits. Based on the results from urethral morphology, tissue reconstruction, luminal patency, and complication incidence (including stone formation, urinary fistula, and urethral stricture) among the animals, it is clear that PLLA/PEG scaffolds combined with hAMSCs led to the best repair of urethral defects. Moreover, histological evaluations show that while the cell-free scaffolds prevented re-epithelialization, hAMSCs facilitated re-epithelialization over the scaffolds. Findings from this study indicate that tissue engineering is a promising approach for urethral regeneration. Certainly, there are also limitations in this study. For example, the mechanisms of hAMSCs-based urethral regeneration remain to be elucidated. In addition, while this study examined the short-term effects of using the combination of hAMSCs and PLLA/PEG nanofibrous scaffolds on urethral repair, long-term studies should be performed following studies. Comparison of the effect of hAMSCs and other types of MSCs on urethral repair is also an area of interest.

## Figures and Tables

**Table 1:** The incidences of post-operation complications including urethral stricture and urinary fistula in rabbits.

Group	Number of Urethral Stricture and Urinary Fistula		Total Number	Incidence (%)
	+	−		
A	0	18	18	0.0
B	1	17	18	5.6
C	13	5	18	72.2

## Abbreviations

ADMSCs	adipose-derived mesenchymal stem cells
α-MEM	α-Mininum essential medium
ANOVA	analysis of variance
BMSCs	bone marrow mesenchymal stem cells
CCK-8	Cell Counting Kit-8
DMEM	Dulbecco’s Modified Eagle’s medium
ECM	extracellular matrix
EDTA	ethylenediaminetetraacetic acid
FBS	fetal bovine serum
IACUC	Institutional Animal Care and Use Committee
IBMX	isobutylmethylxanthine
ITS	insulin-transferrin-selenious acid mix
hAMSCs	human amniotic mesenchymal cells
H&E	hematoxylin-eosin
HRP	horseradish peroxidase
MSCs	mesenchymal stem cells
PCL	polycaprolactone
PEG	poly(ethylene glycol)
PGA	polyglycolic acid
PLA	polylactide
PLGA	poly(l-lactide-*co*-glycolide)
PLLA	poly(l-lactide)
RUG	retrograde urethrogram
SEM	scanning electron microscopy
